# A biologging database of mobulid rays from the Gulf of California, Mexico

**DOI:** 10.1038/s41597-023-02874-w

**Published:** 2024-01-04

**Authors:** Kelly M. Zilliacus, John O’Sullivan, Felipe Galván-Magña, Megan K. McKinzie, Donald A. Croll

**Affiliations:** 1 Department of Ecology and Evolutionary Biology, University of California Santa Cruz, Santa Cruz, California, 95060 USA; 2 Monterey Bay Aquarium, Monterey, California, 93940 USA; 3 Instituto Politécnico Nacional, Centro Interdisciplinario de Ciencias Marinas, La Paz, Baja California Sur Mexico; 4 Monterey Bay Aquarium Research Institute, Moss Landing, California, 95039 USA

**Keywords:** Animal migration, Conservation biology

## Abstract

We initiated a tagging program in 2004 to determine the large-scale and long-term movement patterns of three species of Mobulid Ray (*Mobula mobular, M. munkiana, M. thurstoni*). Between 2004 and 2014 we deployed 48 pop-up archival (PAT) tags that recorded temperature, pressure, and light level. Pressure and light level records were then used to calculate animal depth and geolocation. Transmitted and when available recovered raw data files from successful deployments (n = 45) were auto-ingested from the manufacturer into the United States Animal Telemetry Network’s (ATN) Data Assembly Center (DAC). Through the ATN DAC, all necessary metadata were compiled, dataset was prepped for release, and derived geolocation trajectories (n = 43) were visualized within their public facing data portal. These data and the full metadata records are available for download from the ATN portal as well as permanently archived under the DataONE Research Workspace member node.

## Background & Summary

Mobulid Rays (family Mobulidae) are pelagic elasmobranchs distributed globally in tropical and warm-temperate waters^1^. They have very low fecundity and are targeted in small scale fisheries as well as captured as bycatch in large scale industrial fisheries making them exceptionally vulnerable to overexploitation^2^. In addition, their large-scale and long-term movement patterns are generally unknown^3^. While numerous tagging studies of elasmobranchs exist, few are focused on Mobulid Rays, with even fewer focused on the smaller devil ray species^3–6^. In an effort to elucidate these patterns to inform fisheries managers and conservation efforts, we initiated a tagging program in June 2004 within the southern Gulf of California, Mexico. The Gulf of California is home to five of the nine Mobulid Ray species (*Mobula birostris*, *M. mobular*, *M. munkiana*, *M. tarapacana*, and *M. thurstoni*), with *M. munkiana* listed as Vulnerable by the IUCN Red List and the other four species listed as Endangered^7^. We focused our efforts on the three most abundant species in the region, *M. mobular*, *M. munkiana*, and *M. thurstoni*. We present here the dataset from the electronic tags applied to these three species. The tags recorded temperature, as well as pressure and light-level data that allow for depth and location to be calculated. All of these data and related metadata are now publicly available through the United States (US) Animal Telemetry Network (ATN) Data Assembly Center (DAC), part of the National Ocean Service (NOS) National Oceanic and Atmospheric Administration (NOAA) Integrated Ocean Observing System (IOOS).

## Methods

### Tagging deployments and study subjects

**Fig. 1:**
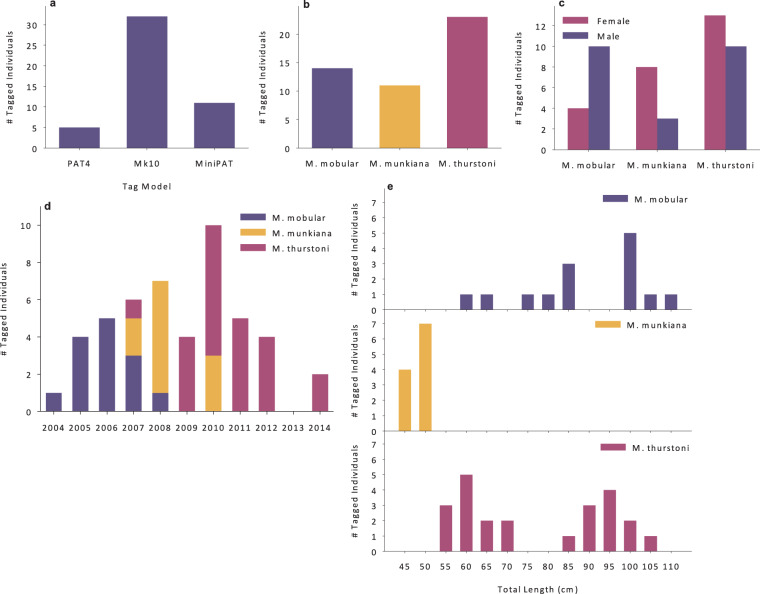
Metadata and deployment summaries of the Mobulid Ray tagging program. (**a**) Types of PAT tags deployed on Mobulid Rays; two-thirds of tags deployed were Wildlife Computers MiniPAT tags. (**b**) Number of tagged individuals by Mobulid Ray species: 14 (29%) *M. mobular*, 11 (23%) *M. munkiana*, and 23 (48%) *M. thurstoni*. (**c**) Sex distribution of tagged individuals by Mobulid Ray species. More male *M. mobular* (71%) were tagged than *M. munkiana* (27% male) or *M. thurstoni* (43% male). (**d**) Deployments by year by Mobulid Ray species. Tag deployments occurred during the month of June in all years except 2014 when tag deployments occurred during March. There were no deployments in 2013. (**e**) Total body length (cm) binned in 5 cm increments for each Mobulid Ray species.

Between 2004 and 2014, 48 *Mobula spp*. were tagged near Isla El Pardito in the Gulf of California, Mexico (14 *Mobula mobular*, 23 *M. thurstoni*, and 11 *M. munkiana*) with Wildlife Computers, Inc. (Redmond, WA) pop-up archival transmitting tags (PAT tags; also known as PSATs – pop-up satellite archival transmitting tags). The tag deployment metadata file contains details on each of the 48 tag deployments including information on the type of tag deployed (model, Platform Transmitter Terminal identification), individual *Mobula spp*. tagged (species, unique identifying number, sex, size), and capture event details (time, date, location) (Tables 1, 2). Tag deployment and Mobulid Ray demographic details are summarized in Fig. 1.

**Table 1:** Metadata descriptions of the tag deployment and *Mobula spp*. details for all tags included in the database.

TagModel	Wildlife Computers PAT tag model: “PAT4”, “Mk10”, “MiniPAT”
TagSerialNumber	PAT tag unique serial number
TagCalibrationDate	Date PAT tag was calibrated in UTC
Sensors	PAT tag sensors
TagIDType	PAT tag ID type: “ptt_id”
TagID	PTT (Platform Transmitting Terminal) ID
ArgosProgramNumber	Argos program number associated with the reported tag ID
DeploymentID	Unique manufacturer assigned identifier for the deployment of a tag on an animal, “NA” indicates metadata were not assigned or available
PATDataTrans	Did the PAT tag transmit data via satellite connection? “Yes” or “No”
DeploymentStartDateTime	Date and time of when an instrumented animal was released, indicating the start of the deployment, in UTC
DeploymentEndDateTime	Date and time of when an instrumented animals’ deployment ended, in UTC
DeploymentDays	Length of tag deployment in days
DeploymentLocation	Name of the location where the instrumented animal was released
DeploymentStartLatitude	The latitude of the location where the instrumented animal was released, in decimal degrees, WGS84 reference system
DeploymentStartLongitude	The longitude of the location where the instrumented animal was released, in decimal degrees, WGS84 reference system
DeploymentEndLatitude	The latitude of the location where the PAT tag first reported its location to the ARGOS system, in decimal degrees, WGS84 reference system
DeploymentEndLongitude	The longitude of the location where the PAT tag first reported its location to the ARGOS system, in decimal degrees, WGS84 reference system

The file, Mobulids_metadata.csv, can be found on the ATN DAC portal as well as the DataONE repository.

**Table 2:** Continuation of metadata descriptions of the tag deployment and *Mobula spp*. details for all tags included in the database.

AnimalCommonName	Common name of the animal on which the instrument was deployed, as defined by the World Register of Marine Species (WORMS, http://www.marinespecies.org/)
AnimalScientificName	Scientific name of the animal on which the instrument was deployed, as defined by the World Register of Marine Species (WoRMS, http://www.marinespecies.org/)
AnimalAphiaID	A taxonomic identifier. This is a Life Sciences Identifier (LSID), a persistent globally unique identifier for the scientific name of the tagged animal, matches the species id reported within the World Register of Marine Species (WoRMS) system (http://www.marinespecies.org/)
AnimalID	Identification code that uniquely identifies each animal, as specified by the researcher
AnimalLength	Length of animal at the time of instrument attachment
AnimalLengthUnits	Units used in animal length field, metric
AnimalLengthType	Type of length measurement reported in animal length field.
AnimalLength2	Secondary length measurement, “NA” indicates metadata were not assigned or available
AnimalLength2Units	Units of secondary length measurement, metric, “NA” indicates metadata were not assigned or available
AnimalLength2Type	Secondary length type, “NA” indicates metadata were not assigned or available
AnimalSex	Sex of tagged animal: “male”, “female”
AnimalLifeStage	Life stage of animal at time of tagging: “unknown”, “adult”, “mature”
Comments	Any researcher notes or additional comments about the deployment

The file, Mobulids_metadata.csv, can be found on the ATN DAC portal as well as the DataONE repository.

**Fig. 2:**
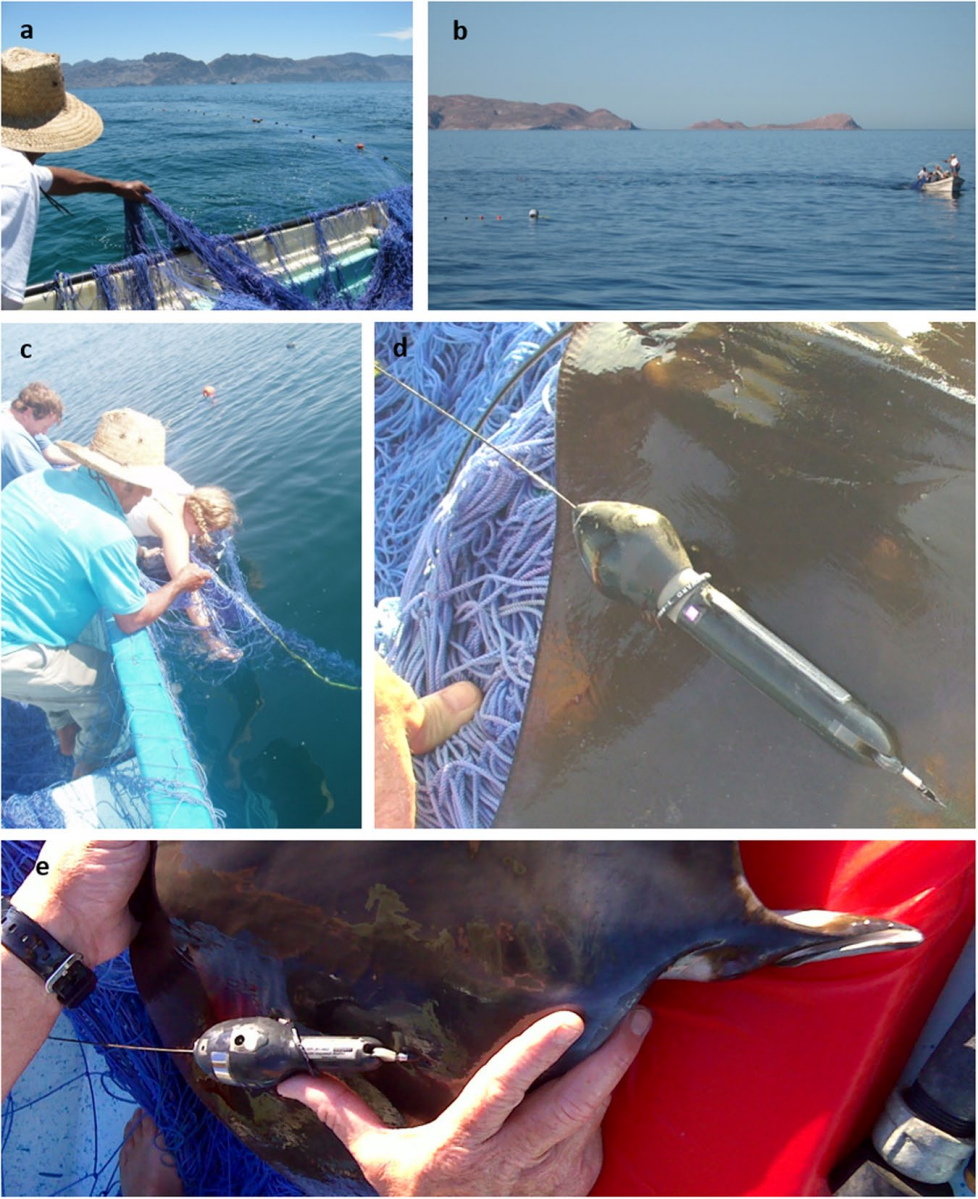
Typical *Mobula spp*. tagging operation in the Gulf of California, Mexico. (**a**) Braided nylon surface net being deployed from a skiff. (**b**) The capture net floating at the surface as the skiff encircles the *Mobula spp*. (**c**) Research team measuring *Mobula mobular* while it is held in the net alongside the skiff. (**d**) Successfully applied Mk10 PAT tag with secondary loop at the base of the float. Size and shape of PAT4 tags are the same as Mk10 PAT tags. (**e**) Successfully applied MiniPAT tag with secondary loop at the base of the float. All participants consent to having their picture taken and used in this manuscript.

Tagging methods are detailed in Croll *et al*.^3^, but are summarized here. *Mobula spp*. were encircled with a 150 m long, 15 m deep, 25 cm mesh braided nylon surface net (Fig. 2a,b). Once captured, *Mobula spp*. were held in the water alongside the skiff to allow for water to flow through their gills (Fig. 2c). Individuals were measured for length and width or half-width (depending on species) and sexed prior to tag attachment. Tags were attached to the dorsal surface along the pectoral fin margin with an aluminium pole and a medical-grade plastic umbrella dart (Fig. 2d). A secondary attachment loop was used to keep the tag flush with the surface of the animal (Fig. 2d).

### PAT platform sensors and configuration

Pop-up archival transmitting (PAT) tags deployed on *Mobula spp*. included PAT4, Mk10, and MiniPAT tags from Wildlife Computers, Inc. (Redmond, WA.). All PAT tag models included wet/dry, light level, pressure, and temperature sensors. Tags were programmed to collect external temperature, depth, and light level data while deployed on *Mobula spp*., and set to release from their anchor approximately 6 months post deployment or after remaining at a constant depth range (within 4–8 m) for 48–96 hours (depending on deployment year). Once released from the *Mobula spp*. tags floated to the surface and transmitted a subset of data to the Argos satellite system. PAT tag post-release transmission details are described in detail in O’Sullivan *et al*.^8^. PAT tags were programmed to prioritize which data to transmit and in what format (Table 3). The full archival dataset was downloaded from any recovered tags providing fine scale temperature, depth, and light level data recorded by the tag.

**Table 3:** Programmed PAT tag metadata descriptions.

AnimalID	Identification code that uniquely identifies each animal, as specified by the researcher
TagModel	Wildlife Computers PAT tag model: “PAT4”, “Mk10”, “MiniPAT”
TagSerialNumber	PAT tag unique serial number
TagID	PAT tag PTT (Platform Transmitting Terminal) ID
DeploymentID	Unique manufacturer assigned identifier for the deployment of a tag on an animal, “NA” indicates metadata were not assigned or available
PATRecovery	Was the PAT tag physical recovered? “Yes” or “No”
PATDataTrans	Did the PAT tag transmit data via satellite connection? “Yes” or “No”
DATABinned	Were the PAT tags programmed to transmit temperature and depth data in pre-determined numerical bins? “Yes” or “No”
DATATS	Were the PAT tags programmed to transmit temperature and depth data as a time series? “Yes” or “No”
SamplingIntervalArchive	PAT tag sampling interval (in seconds) for temperature and depth (this may be different from the interval between observations in the time series file if the transmitted data were thinned)
DataBinsHrs	Duration (in hours) of each data bin for constructing the depth and temperature histograms
UTCOffsetHrs	Offset in hours from UTC for the start of the histogram data, “NA” indicates no offset
TempBins	Break points for the temperature bins for the histograms
DepthBins	Break points for the depth bins for the histograms

The file, PAT_programming_table_Mobulids .csv, can be found on the ATN DAC as well as the DataONE repository.

Light level data was used to estimate the location of the *Mobula spp*. by producing two light-level curves each deployment day as described by O’Sullivan *et al*.^8^. The light level curves are used to produce geolocation estimates (latitude and longitude) and are dependent on the quality of the light curves^8^. Wildlife Computers proprietary geolocation algorithm, GPE3, was used to process the light-level data to further refine the geolocation estimates. The GPE3 model runs contain two types of uncertainty around the geolocation estimate, observation light level mean sum of squares and model score (more information can be found at www.wildlifecomputers.com). Users are required to enter the estimated swim speed of the tagged animal, as well as the deployment start and end locations. For each tagged Mobulid ray, we conducted multiple runs of the GPE3 software using 0.75 ms^−1^, 1.0 ms^−1^, 1.25 ms^−1^, 1.5 ms^−1^, and 2.0 ms^−1^ for animal swim speed. Overall, we found the model runs at a swim speed of 1 ms^−1^ to have the highest overall model scores, and thus we exported those model runs to the ATN DAC and DataONE repository.

### Data transmission and processing

Data from successful PAT tag deployments (n = 45) were transmitted to Wildlife Computers through Argos Services and decoded using the Wildlife Computers data analysis program (DAP; Wildlife Computers, Inc.). Archival data from recovered tags (n = 7) were manually uploaded directly to the Wildlife Computers data portal and decoded using the DAP. Similar to O’Sullivan *et al*.^8^, decoded raw telemetry data and processed GPE3 files were then downloaded from the Wildlife Computers data portal to the ATN DAC via the Wildlife Computers API as .csv files and in some cases in the proprietary WC file format using the unique manufacturer assigned deployment identifier (Tables 1,2). Downloaded data were zipped and maintained as is.

## Data Records

Unique identification numbers assigned by researchers (i.e., AnimalID) were used to label each zip file (see Table 2). Similar to O’Sullivan *et al*.^8^, the subset of files included within each deployment folder are contingent on tag model, programming selections and whether a tag was successfully recovered. Transmitted and if available, recovered data were merged prior to release to the ATN DAC. Individual data files, regardless of tag type, were labelled using the tag’s assigned Platform Transmitter Terminal (PTT) id and the specific WC file type. Processed GPE3 files are labelled using animal id and the number of the selected GPE3 file run. To assist with future merging and reuse of these data, unique deploy id (i.e., AnimalID), PTT ID and tag type were included within each individual data file.

Full data records and metadata from the 45 tags deployed on *Mobula spp*. from 2004–2014, as well as an ISO 19115 metadata record with geospatial data, are publicly available through the Research Workspace (RW) Data Observation Network for Earth (DataONE) member node (https://search.dataone.org/portals/RW) as well as the ATN data portal (https://tinyurl.com/3k44ca4e) where the location files (i.e. GPE3-X.csv) are also visualized. These data have a standalone, upstream Digital Objective Identifier (10.24431/rw1k7du) specific to the dataset itself^9^ and a standard CC-BY license. These data and free to use without restriction, however, we request that future users acknowledge the ATN as well as cite this data manuscript in any representations of the data and/or future publications. The *M. mobular* data were previously published in Croll *et al*.^3^.

## Technical Validation

### Post-processing of raw data

Similar to O’Sullivan *et al*.^8^, raw data files were exported directly from the tag manufacturer (Wildlife Computers) by the ATN DAC and preserved as is. Files were reviewed by ATN for completeness and to ensure the correct labels were applied to files and folders, and proper ids were provided. We strongly encourage new users to read and fully comprehend associated metadata, and data files prior to use.

## Usage Notes

Three of our tagged *M. thurstoni* (Mx07_29_MTh, Mx11_02_MTh, and Mx14_03_MTh) did not report any data and were thus excluded from the archived dataset. In addition, two of our tagged *M. thurstoni* (Mx10_19_MTh and Mx14_02_MTh) did not report enough data to run the GPE3 location process.

## Data Availability

No custom code was used to generate or process the data described in this manuscript.
